# Should the breast be a check area on standard CT thorax examinations?

**DOI:** 10.1186/bcr3310

**Published:** 2012-11-09

**Authors:** NL Marshall, L Duddy, J Barry, R Murphy, P Smiddy, M Ryan, D Hill

**Affiliations:** 1 University College Cork, Ireland

## Introduction

With increasing cross-sectional imaging, incidental findings (IF) are common across all subspecialities. There are implications for patients and the health service consequent to IFs: anxiety, further radiation and biopsy. Our purpose was to evaluate the malignant assessment (MA) rate for patients referred to the symptomatic breast clinic after a breast IF and to compare this with the general MA rate.

## Methods

This retrospective review spanned 2 years. A trawl was performed of breast imaging reports for CT-detected lesions. Demographics, imaging and pathology results were collated. Statistical analysis using Fisher's exact test was performed to identify demographic factors associated with MAs. The MAs for the IF cohort were then compared with the general clinic population using chi-square analysis.

## Results

There were 103 patients with IFs (102 women, one man) comprising 49 lesions (30 benign, 19 malignant). In the general service were 10,330 patients with 2,551 lesions (683 malignancies). The IF cohort were more likely to have a lesion (47.6% vs. 28.7%, *P *< 0.001) and to have a MA (18.4% vs. 6.7%, *P *< 0.0001). The only demographic factor of the IF group with a statistically significant association with MA was age >65 years (*P *= 0.0063).

## Conclusion

While patients with breast IFs are more likely to have a MA, only 47% of those referred actually had a lesion. Age was the only statistically significant factor that correlated with a MA. Whilst a worthy check area clinicians should exercise caution to avoid unnecessary procedures, particularly in younger patients.

